# Heterogeneous Network Model to Identify Potential Associations Between *Plasmodium vivax* and Human Proteins

**DOI:** 10.3390/ijms21041310

**Published:** 2020-02-15

**Authors:** Apichat Suratanee, Kitiporn Plaimas

**Affiliations:** 1 Department of Mathematics, Faculty of Applied Science, King Mongkut’s University of Technology North Bangkok, Bangkok 10800, Thailand; apichat.s@sci.kmutnb.ac.th; 2 Advanced Virtual and Intelligent Computing (AVIC) Center, Department of Mathematics and Computer Science, Faculty of Science, Chulalongkorn University, Bangkok 10330, Thailand; 3 Omics Sciences and Bioinformatics Center, Faculty of Science, Chulalongkorn University, Bangkok 10330, Thailand

**Keywords:** *Plasmodium vivax*, iterative network propagation, heterogeneous networks, malaria, host-malaria interactions, network analysis

## Abstract

Integration of multiple sources and data levels provides a great insight into the complex associations between human and malaria systems. In this study, a meta-analysis framework was developed based on a heterogeneous network model for integrating human-malaria protein similarities, a human protein interaction network, and a *Plasmodium vivax* protein interaction network. An iterative network propagation was performed on the heterogeneous network until we obtained stabilized weights. The association scores were calculated for qualifying a novel potential human-malaria protein association. This method provided a better performance compared to random experiments. After that, the stabilized network was clustered into association modules. The potential association candidates were then thoroughly analyzed by statistical enrichment analysis with protein complexes and known drug targets. The most promising target proteins were the succinate dehydrogenase protein complex in the human citrate (TCA) cycle pathway and the nicotinic acetylcholine receptor in the human central nervous system. Promising associations and potential drug targets were also provided for further studies and designs in therapeutic approaches for malaria at a systematic level. In conclusion, this method is efficient to identify new human-malaria protein associations and can be generalized to infer other types of association studies to further advance biomedical science.

## 1. Introduction

*Plasmodium vivax* (*P. vivax*) is one of the five species of *Plasmodium* that affect humans. It is highly prevalent in most areas in Latin America and Southeast Asia [1] and people of all ages are at risk for this parasite [2]. *P. vivax* has a dormant stage in human liver, where it forms hypnozoites during its life cycle allowing the sporozoite to survive inside the liver cells [3]. This causes no symptoms and is undetectable in blood tests, which can cause relapse ranging from weeks to years after the infection. *P. vivax* is a cause of morbidity and mortality in young infants who are infected with the malarial parasite, and is also associated with a high risk of severe anemia in the young infants [4]. Moreover, infection with *P. vivax* during pregnancy increases the risk for miscarriage and reduced birth weight [5].

The method of treating *vivax* malaria is to cure at the blood infection stage by eradicating hypnozoites from the liver to prevent relapses. Artemisinin-based combination therapy (ACT) is found to be highly effective against *P. vivax* malaria [6,7]. Therefore, ACTs have been used to treat the blood stage of all malaria. Specifically to *vivax* malaria, chloroquine is an effective treatment in many areas but the resistance of *P. vivax* to chloroquine frequently arises with the highest risk in Indonesia [8]. With signs of artemisinin resistance emerging, strong gains were made through combinations such as artemisinin-combination therapies (ACTs), a process of combining artemisinin with a partner to combat resistance.

For treating the liver stage of infection, primaquine is used for preventing relapse. However, primaquine can destroy red blood cells in people with a hereditary deficiency of the glucose-6-phosphate-dehydrogenase enzyme (*G6PD*). Therefore, primaquine was avoided in areas where the population commonly have this deficiency [9]. This safety concern resulted in impeded widespread use of primaquine. In addition, the optimum duration of treatment and best partner drug are uncertain [10]. It was found that the periodical relapse of tropical stains of *P. vivax* requires a higher primaquine dose for radical cure than for the strains found in other places [11].

Recently, a new radical drug cure, tafenoquine, has become available as a single dose. Single-dose tafenoquine resulted in a significantly low risk of *P. vivax* recurrence as compared with a placebo in patients with normal *G6PD* activity [12]. However, tafenoquine can cause hemolysis in patients with *G6PD* deficiency. Therefore, *G6PD* diagnostic testing is still recommended to determine eligibility for tafenoquine use [12,13,14].

Enhancing the radical drug cure with the potential to significantly reduce relapse infection and drug resistance is a challenging task. To accomplish this task, we need to better understand malaria’s ability to evade the human immune system and recruit host responses to regulate its life cycle. These processes lead to malaria to acclimatize to the host environment. Therefore, the understanding of vivax malaria and human protein interactions within the human host during infections is important to develop strategies to cure this infectious disease. Several studies have increasingly lead to the perception that interactions between *P. vivax* proteins and host factors are essential to establish the infection and virulence at every stages of the malaria life cycle [15]. However, maintaining a *P. vivax* continuous culture in vitro is extremely difficult, resulting in major delays in developing an effective functional malaria vaccine [16,17]. Identifying each parasite protein’s function in the complex process of *P. vivax* invasion and evaluating new therapeutic agents are still the challenging tasks. Therefore, computational methods could be employed to identify novel drug targets to develop new alternative drugs to treat malaria.

The analysis of protein interactions has been performed for many species; especially, in humans and bacteria [18,19,20,21,22]. This type of analysis is rarely found in malaria studies and almost all the analysis has been performed on *Plasmodium falciparum*. The research began in 2005 with the work of LaCount et al. in identifying *P. falciparum* protein-protein interactions using a high-throughput technique of the yeast two-hybrid approach [23]. In 2012, an inhibition analysis of protein-protein interactions was performed to find a new drug target [24]. In 2014, Liu et al. proposed a new way to predict protein interactions related to the invasion of erythrocytes by malarial parasites [25]. Recently, the analysis of the parasite-human protein interaction was performed on a protein target or agent having an association with a human host [26,27]. At present, Hillier et al. developed a landscape of the *Plasmodium* interactome to find out conserved and species-specific functionality among various *Plasmodium* species [28]. Not only has some analysis of the malaria data at the protein interaction level has been performed, but also at metabolic network level has been performed for identifying drug targets in *Plasmodium* [29,30]. Note that all analyses mentioned here were performed on *Plasmodium falciparum*. There is a lack of studies that analyze *Plasmodium vivax* with more sophisticated computational approaches like network analysis and modeling.

**Figure 1:**
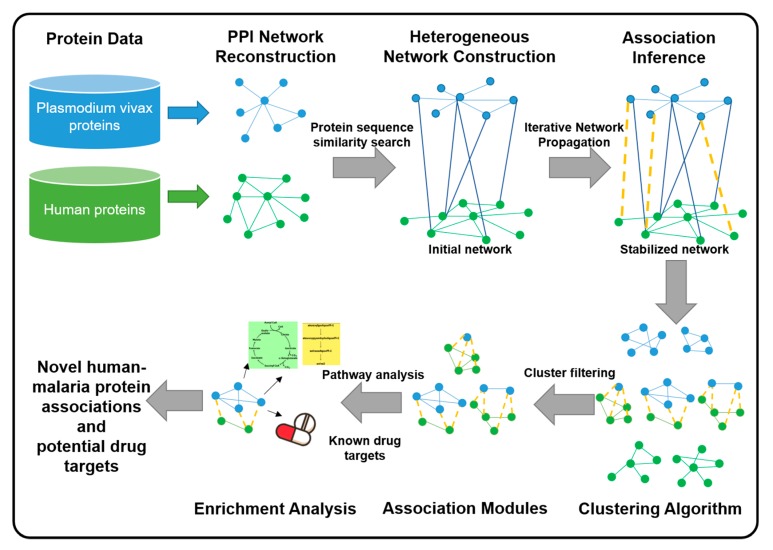
The meta-analysis framework with the use of a heterogeneous network model for inferring human-malaria protein associations.

In this study, we propose a novel meta-analysis framework with the use of a heterogeneous network model by integrating protein interaction networks between humans and *Plasmodium vivax* parasite into one association network, as shown in Figure 1. The full network consists of two different types of nodes: human proteins and malaria proteins. Protein-protein relationships of humans as well as those of malaria are constructed based on protein interaction information from the STRING database [31], Copenhagen, Denmark; Heidelberg, Germany; Lausanne, Switzerland. Human–malaria relationships are initiated based on protein sequence similarities. The filtering of novel human-malaria protein association is thus formulated as a prioritization problem of human-malaria protein pairs on this heterogeneous network. An iterative updating algorithm that propagates information across the network is then applied to score the existing edges and to fill the missing edges until a stabilized network is obtained. All proteins on this stabilized network are then clustered into association modules. Each module is then examined for enrichment in protein complexes and drug target information from the existing databases CORUM [32] (München, Germany) and DrugBank [33] (Edmonton, Alberta, Canada), respectively. Our meta-analysis framework with the use of the heterogeneous network automatically incorporates protein interactions and sequence similarities into human-malaria protein association prioritization. After that, cluster analysis filtering is applied, and the pathway enrichment is performed. Thus, some promising associations are found and reported with protein complexes and drug target associations.

## 2. Results

### 2.1. Initial and Stabilized Heterogeneous Networks

We obtained 1787 *Plasmodium vivax* malaria proteins and 12,038 human proteins which have at least an edge connecting between the same type of proteins. Among malaria proteins, there were 11,477 connections with high confidence scores of reliable protein interactions; while for human proteins, there were 313,359 protein interactions with the same criteria. With the number of obtained malaria and human proteins in their own interaction networks, there were 21,511,906 possible connections between these two types of protein nodes. Since the true connections across these two species are quite rare and yet to be determined, the initial connections among these two types of nodes were determined by protein homologs with the protein sequence similarities. All *P. vivax* protein sequences were searched against all human protein sequences. Consequently, we assumed 9525 links between malaria proteins and human proteins, and we could firstly establish a two-layer heterogeneous network consisting of malaria protein pairs, human protein pairs, and human-malaria protein pairs, which were obtained from malaria protein interactions, human protein interactions, and human-malaria protein similarities, respectively. However, the network at the initial state is unlikely to comprise all possible true associations as there are some missing edges and overweighed edges for the human-malaria protein associations. Thus, further analysis was required to iteratively update the edge weights from the initial state until the network is stabilized. To see an overview of all networks, Table 1 represents the number of nodes and edges, and the properties of human protein interaction, malaria protein interaction, and the heterogeneous networks with initial human-malaria proteins connections and after the network propagation until it stabilized.

Notice that, in Table 1, there were 21,511,906 pairs of human and malaria proteins (12,038 human proteins × 1787 malaria proteins). It is quite a high number, and our final stabilized network could filter approximately 28% out of the total pairs, which were 6,422,644 − (313,359 + 11,477) = 6,097,808 pairs. Next, our framework qualified this list with the clustering analysis with protein complexes and known drug targets. Finally, in total, 14,844 human-malaria protein pairs were selected and discussed later in the other sections.

**Figure 2:**
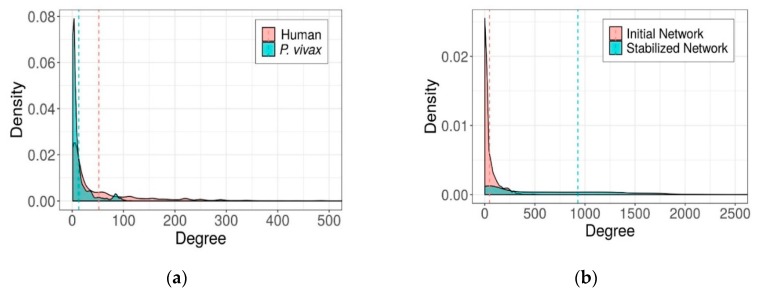
Degree distribution of the established network. (**a**) Degree distributions of human and malaria protein interaction networks. (**b**) Degree distributions of initial heterogeneous and stabilized heterogeneous networks. Dash lines shows the mean values of these distributions.

Comparing the structures of the human network and malaria network separately, we found that their degree distributions were significantly different with *t*-test (*p*-value less than 2.2 × 10^−16^) as shown in Figure 2a. These two networks follow the power-law distribution, which means the networks have many low-degree nodes and small numbers of high-degree nodes. As expected, the malaria network comprises a greater number of low-degree nodes compared to the human network. Average degree of the malaria network was thus less than that of the human network with the values of 12.84 and 52.06, respectively. Figure 2b shows the change in degree distributions of initial and stabilized heterogeneous network. The degree distribution of the initial heterogeneous network also follows the power law distribution, while that of the stabilized network diverges from the power-law distribution and displays a higher density in the network—as expected since there are many more edges connecting two types of nodes which were recovered by the network propagation algorithm. Biologically, protein similarities between humans and malaria reflect homologs and infer to be responsible for the same cellular functions. However, to survive and take advantage of the human host, these homologous proteins in malaria might be a connection to regulate the other important genes in humans to maintain their lives in the host. Therefore, the interacting proteins to these homologs could be able to interact or even work together to maintain the development functions as well. Note that after performing the network propagation, the stabilized network might contain all possible links. However, all these links had their own weights of confident associations between human and malaria proteins. The higher the obtained weight of the link; the more the confidence of the associations was rated. Therefore, our final stabilized network was obtained when we set the cutoff threshold of the weight that yielded the best performance to obtain human-malaria protein association. After that, these resulting human-malaria protein pairs were clustered and evaluated by further analyses with protein complexes and drug targets.

### 2.2. Performance in Identifying Human-Malaria Protein Associations

**Figure 3:**
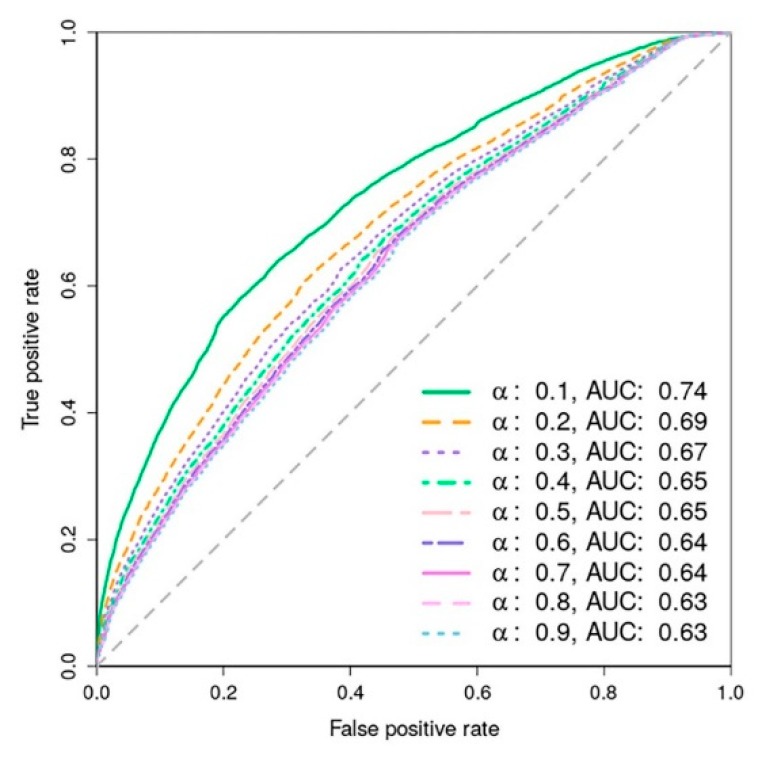
Receiver operating characteristic (ROC) curves of different values of parameter α.

To determine the performance of the framework algorithm, we performed ten times ten-fold cross-validations to evaluate the performance in terms of accuracy of the heterogeneous network filtering (more detailed in Section 4). All human proteins were randomly partitioned into ten parts. For each part, we removed weights of all human-malaria protein connections whose human proteins were in the discarded part. After that, the remaining human-malaria protein pairs were used to recalculate their edge weights by the iteratively updated method. When the stabilized network was obtained, the weights of the removed human-malaria protein pairs were collected as their association scores and then compared with their sequence homology search results. The different thresholding on association scores for filtering the list of the possible associations was performed. Then, the standard true-positive rate against the false-positive rate was measured as a receiver operating characteristic (ROC) curve and the area under the curve (AUC). The average performance of these ten-time cross-validations was calculated to get the overall accuracy of the filtering. Based on the propagation algorithm to obtain a stabilized network, there was an important factor known as a decay factor (α) to diffuse the initial weights of human-malaria proteins to the other missing edges. Thus, the performance was measured for all possible values of parameter α from 0.1 to 0.9 in steps of 0.1, as shown in Figure 3. Notice that the performance gradually declined when the value of α was increased. The best performance was found at the low alpha parameter (α = 0.1) with an area under the ROC curve of 0.74.

By maximizing the sensitivity and specificity of the best performance curve (α = 0.1), the best weight cutoff to determine whether a connection should be defined or not was found at 0.0003. If an association score of a connection is greater than this cutoff value, the connection is established. With this criterion, we yielded the sensitivity of 0.75 and specificity of 0.60. This resulted in 6,097,808 obtained human-malaria protein pairs (28.34% out of all possible pairs) for our stabilized heterogeneous network.

To demonstrate the advantage of the protein interaction networks, we further performed a test by creating many random networks, where the protein’s labels of the human network and those of the malaria network were randomly shuffled when maintaining the networks’ structures as their original structures since the edges were not changed. The network propagation algorithm was then applied to these random networks using the same initial human-malaria protein associations derived from the sequence similarity searches. The random experiments were repeated three times. For each repetition, we conducted the same procedures of the performance evaluations. With the optimal alpha values of 0.1, we obtained the best performance of the AUC from all experiments of 0.55, which showed very low performance. This indicates that the information of both human and malaria protein interaction networks is crucial and useful information for inferring human-malaria protein associations from the heterogeneous network and the network propagation algorithm.

### 2.3. Association Modules and Protein Complexes on the Heterogeneous Network

The stabilized heterogeneous network with human and malaria protein interaction network was then clustered to find modules of dense connections using the ClusterONE algorithm [34]. With this clustering algorithm, we obtained 970 module clusters (as provided in Supplementary Table S1). To select only core clusters, we examined whether the human proteins in each cluster were overrepresented in protein complexes. To achieve this purpose, we employed protein complex data from the CORUM database [32], München, Germany. The enrichment test was performed for every network module. Approximately 387 clusters were found to be formed as a protein complex with the Benjamini-Hochberg corrected *p*-values of their enrichment tests < 0.01 (The list of Cluster ID, sets of enriched protein complexes, and their *p*-values can be found in Supplementary Table S2). Note that some groups of proteins, known as our network modules, were enriched for more than one group of protein complexes.

For each module cluster in which their human proteins were enriched in protein complexes, the associations between human proteins and malaria proteins in the clusters were collected. Note that some clusters have either only human proteins or only malaria proteins. However, this study aimed to select more reliable human-malaria protein associations. Therefore, only module clusters containing both types of proteins and their retrieved associations were selected. We then obtained 1,050,701 human-malaria protein associations from all clusters, as reported in Supplementary Table S3. With these 1,050,701 associations from all clusters, there are totally 497,815 unique associations. This is the first established list of potential human-malaria protein associations to be further analyzed and tested either computationally or experimentally and to be collected in a database. Furthermore, with this data set, we can then choose the top list of the associations by varying the association scores for further analyses of our interest. In this study, we qualified this list further with potential drug targets to cure the *Plasmodium vivax* malaria.

### 2.4. Identification of Potential Drug Targets in Humans to Treat Malaria

To identify potential drug targets from the resulting human-malaria protein associations, the association modules or clusters that were protein complexes were then analyzed for known drug targets. From 387 clusters enriched with protein complexes, we found 24 clusters enriched in drug targets (reported in Supplementary Table S4). Out of these clusters, there were 7 clusters that also contained malaria proteins, with the rest of the clusters having only human proteins. All seven clusters—with their Cluster IDs, the number of human-malaria protein associations, the number of known drug targets, the number of unknown drug targets, and the ratio of known drug targets—are shown in Table 2, where a star (*) marks the clusters that have the ratio of human proteins that were known drug targets to all human proteins in the cluster of more than 0.6. With these clusters, 41,549 human-malaria protein associations were found (Supplementary Table S5). With 41,549 associations from all clusters, there are totally 41,477 unique associations, of which 14,844 are pairs whose human protein is a known target for an approved drug in the DrugBank database, Edmonton, Alberta, Canada. The list of proteins and the cluster pictures for these seven clusters can be found in Supplementary Table S6 and Supplementary Figure S7, respectively.

**Figure 4:**
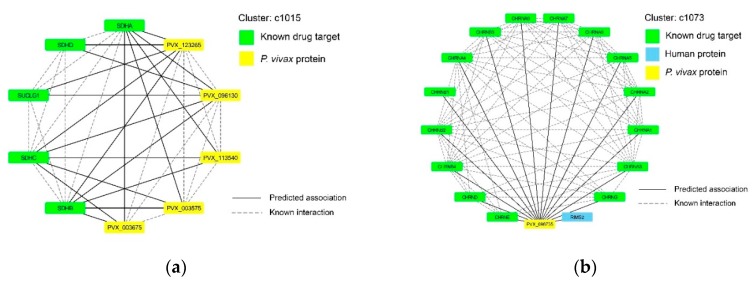
Two selected clusters that contain more than 90% of known drug targets. (**a**) Cluster c1015 consists of 19 associations of five human proteins (*SUCLG1*, *SDHA*, *SDHB*, *SDHC*, and *SDHD*) and five malaria proteins (*PVX_096130*, *PVX_123265*, *PVX_003675*, *PVX_113540*, and *PVX_003575*). (**b**) Cluster c1073 consists of 16 associations of sixteen human proteins and only one malaria protein (*PVX_098735*).

Interestingly, Clusters c1015 and c1073 obtained a high ratio of known drug targets in humans, as shown in Table 2 and Figure 4. Cluster c1015 contains five malaria proteins associated with five human proteins that were all known drug targets; while Cluster c1073 contains only a malaria protein (*PVX_098735*) associating to sixteen human proteins, fifteen of which are known drug targets. These two were qualified more for their related functions and associated drugs with pathway enrichment analysis and drug-target associations.

#### 2.4.1. Potential Targets of Succinate Dehydrogenase Complex

Succinate dehydrogenase complex is an enzyme complex, which we found in Cluster c1015 (see Figure 4a). All human proteins in this cluster were known to be drug targets and there were 19 associations of these five human proteins (*SUCLG1*, *SDHA*, *SDHB*, *SDHC*, and *SDHD*) and five malaria proteins (*PVX_096130*, *PVX_123265*, *PVX_003675*, *PVX_113540*, and *PVX_003575*). This enzyme complex can be found in many bacterial cells and in the inner mitochondrial membrane of eukaryotes. It is the only enzyme that participates in both the citric acid cycle and the electron transport chain [35]. It consists of four subunits (named A to D) encoded by the nuclear genome. Sequence analysis of the flavoprotein (Fp) subunit of *SDHA* and iron-sulfur (Ip) of *SDHB* were reported to show unique features of the catalytic subunit in *Plasmodium* complex II [36]. We did not find any evidence in scientific literature for an association between *SDHC*, *SDHD*, and *SUCLG1* to *Plasmodium*. This is the first computational approach to qualify the possibility of these associations among human and *P. vivax* malaria proteins. Thus, succinate dehydrogenase complex would be a great target for curing this type of malaria.

These five human proteins were further investigated for the pathways they were involved in. We found 9 related pathways. Obviously, there is only citrate (TCA) cycle pathway in which all these five proteins were present. It was reported that mitochondrial metabolic plasticity is essential for parasite development. *P. falciparum* has significant flexibility in its TCA metabolism [37]. The other enriched pathways are shown in Table 3. Furthermore, we investigated the five *P. vivax* proteins and found that they were enriched in the ubiquinone and other terpenoid-quinone biosynthesis with a *p*-value of 8.1 × 10^−6^.

To find drugs that were related to these five proteins, we used the drug information from DrugBank [33] (Edmonton, Alberta, Canada) and the results are shown in Table 4. In total, these five proteins were related to four drugs—consisting of DB00139, DB04657, DB09270, and DB00756—which are small molecule drugs. For clinical trials, DB00139 has been used for treating unspecified adult solid tumor (protocol specific) (phase 1), postpartum anemia (phase 1, 2), anemias (phase 4), and for preventing cognitive dysfunctions (phase 3). DB04657 or Carboxin is used for targeting succinate dehydrogenase (ubiquinone) flavoprotein subunit, mitochondrial in humans; at present, there are no clinical trials for this drug [33]. DB09270 or Ubidecarenone is called coenzyme Q10 and is a powerful antioxidant [38]. The ubidecarenone is a coenzyme related to mitochondrial enzyme complexes and involved in oxidative phosphorylation in the production of ATP, which is sold as a dietary supplement and is not FDA approved as a drug, however, it is recommended to be used under discretion. For pharmacology, coenzyme Q10 was studied to use for treating of several diseases, e.g., Parkinson’s [39], fibromyalgia [40,41], migraine [42,43], and periodontal disease [44,45]. DB00756 or Hexachlorophene is a bacteriostatic cleansing agent. In clinical trials, it was used for treating human immunodeficiency virus infections and infection caused by staphylococci, acne vulgaris (phase 3), dental pain (phase2), and used for preventing methicillin-resistant staphylococcus aureus skin infections [33]. The complete list of these five proteins with their associated drugs and drug descriptions is provided in Supplementary Table S8.

#### 2.4.2. Potential Targets of Nicotinic Acetylcholine Receptor

Cluster c1073 contains only one malaria protein (*PVX_098735*) and sixteen human proteins (*CHRNA1*, *CHRNA2*, *CHRNA3*, *CHRNA4*, *CHRNA5*, *CHRNA6*, *CHRNA7*, *CHRNA9*, *CHRNB1*, *CHRNB2*, *CHRNB3*, *CHRNB4*, *CHRND*, *CHRNE*, *CHRNG*, and *RIMS2*), which are all involved in neurological functions in humans, especially as subunits of nicotinic acetylcholine receptor. *PVX_098735* is a coatomer subunit beta, which is part of a coatomer complex required for budding from Golgi membranes and for the transportation of dilysine-tagged proteins [46]. It is known that malaria can lead to acute or long-term neurological deficits that cause vascular obstruction and reduce cerebral blood flow [47]. There was an interesting study of Gisselmann et al. [48] on the effects of well-known anti-malaria drugs like Quinine and its derivatives to human nicotinic acetylcholine receptors. Their results showed the effects of the quinolone derivatives quinine, quinidine, and chloroquine on human adult and fetal muscle nicotinic acetylcholine receptors and revealed that the clinically proven efficacy of quinine could be based on targeting nicotinic acetylcholine receptors. It might be possible that nicotinic acetylcholine receptors are related to malaria mechanisms in humans. With our framework analysis, we have found this human receptor related to a protein of *P. vivax*. It could be a good hint for further drug development in this direction. Thus, further analysis with pathway enrichments reveals that neuroactive ligand-receptor interaction, cholinergic synapse and nicotine addiction are highly overrepresented among these sixteen human proteins.

Fifteen of these proteins were known to be targets for existing drugs from DrugBank [33] (Edmonton, Alberta, Canada), and only the protein *RIMS2* has no drug yet. In total, these fifteen proteins were related to 58 approved drugs, which are widely used to treat various symptoms of many diseases. The list of these fifteen proteins with their associated drugs and drug descriptions is provided in Supplementary Table S9.

## 3. Discussion

The meta-analysis framework for identifying human-malaria protein associations is proposed in this article. This framework started at the construction of a two-layer heterogeneous network in which the first layer was the human protein interaction network and the second layer was the *Plasmodium vivax* malaria protein interaction network. The connections of human proteins and malaria proteins was then derived from human-malaria protein similarities. The structures of this heterogeneous network had a power-law degree distribution which means this network has a small number of high-degree nodes and a large number of low-degree nodes. In general, all edges in the network were weighted initially by their protein interaction scores. After that, the network information propagation was performed by an iterative approach for renewing edge weights until the network was stabilized. To measure the performance of the iterative algorithm, we performed ten times ten-fold cross-validations. Association scores for human-malaria protein pairs were determined from the average weights after the stabilization from all repetitions. Comparing these scores to the original defined scores of only human-malaria protein pairs, the ROC curve yielded an accuracy of 74% with the optimal parameters. With these experiments, we obtained the optimal parameter and used it for the final model and then performed the association filtering by the heterogeneous network. There were approximately 6,097,808 (28%) of all possible human-malaria protein pairs firstly filtered as those likely to be human-malaria protein associations.

Since the number of the first filtered associations were quite high at more than 6 million pairs, the clustering algorithm for detecting similar modules whose human proteins might form protein complexes were then applied, and each resulting module was tested for the enrichment of known sets of protein complexes (see Supplementary Table S10). Only modules containing both human and malaria proteins and their connections were selected. Thus, after this procedure, 497,815 human-malaria protein associations were reported and examined further with a set of known drug targets. Finally, in total, 14,844 human-malaria protein pairs were selected from the 24 clusters enriched with known drug targets and we found 7 clusters presenting both human proteins and *P. vivax* proteins. These pairs were proposed as our potential associations between human and *P. vivax* proteins. We investigated human proteins for *P. vivax* malaria-related phenotypes by manual literature curation and OMIM search. We found 11 human proteins consisting of *ACKR1*, *CD36*, *CISH*, *CR1*, *FCGR2B*, *HBB*, *ICAM1*, *NOS2*, *SLC4A1*, *TIRAP*, and *TNF*. Unfortunately, these 11 human proteins were not presented in the filtered 7 clusters. This might be because they are not part of any protein complexes we found. However, we investigated the association scores of these proteins to *P. vivax* proteins and found that these scores were significantly higher than the scores of the rest associations (*p*-value < 2.2 × 10^−16^). This result presented the significance of our association scores to the relationship between known human related proteins and *P. vivax* proteins.

Interestingly, for our potential associations, we found a cluster containing mostly human proteins of succinate dehydrogenase complex, and all the human proteins in this clusters were known drug targets for the treatment of several diseases. In addition, we found the nicotinic acetylcholine receptor, which is important for regulation of synaptic transmission and synaptic plasticity as well as signal transmission for sympathetic and parasympathetic systems in the human central nervous system. These were promising target candidates for drug repurposing in curing malaria as well.

## 4. Materials and Methods

### 4.1. Datasets

With our meta-analysis framework (Figure 1), we employed various information from different data sources. All pairwise protein-protein interactions were then selected with high confidence scores. Initial human-malaria protein associations were obtained from sequence similarities. The datasets in this framework are explained as follows.

#### 4.1.1. Protein Interaction Information

We first downloaded protein interaction information of human and *Plasmodium vivax* malaria from String database version 11 [31], Copenhagen, Denmark; Heidelberg, Germany; Lausanne, Switzerland. To construct its own protein-protein interaction network of each organism, protein nodes were connected when their interaction confidence scores were greater than a threshold of 0.9. For *Plasmodium vivax* proteins, interaction information on gene products encoded in the genome was obtained from this database as well. All their gene identifiers and symbols were mapped using the information dataset from the NCBI database, Bethesda, Maryland, United States. 

#### 4.1.2. Protein Sequence Similarity Information

The total of 5392 protein sequences of *Plasmodium vivax* at all developmental stages were retrieved from KEGG databases [49,50], Tokyo, Japan. These protein sequences were searched against all human protein sequences from NCBI database via BlastP, Bethesda, Maryland, United States, with E-value less than 0.00001. The list of similar human proteins for each malaria protein was obtained and simultaneously transformed into known homologous human-malaria protein pairs. The weights of these similar human-malaria protein pairs were then initially defined as 1 and the dissimilarity pairs were set as 0. These weights were used as initial weights for the iterative propagation processes. Finally, a total of 1787 malaria proteins and 12,038 human proteins which have at least one connection across species were selected to construct the heterogeneous network. Consequently, an overall 9525 possible human-malaria protein pairs were known as initial human-malaria protein associations in the network.

#### 4.1.3. Protein Complex and Drug Target Information

Protein complexes were downloaded and extracted from CORUM database [32], München, Germany. To find proteins that are associated with diseases and importantly interactive for drugs, the drug and target information were obtained from the DrugBank database (version 5.1.2) [33], Edmonton, Alberta, Canada. Only approved drugs and their targets were selected. In total, there were 83.61% (2158) of all drug targets in this database found to be human proteins, 7.17% (185) drug targets were *Escherichia coli* proteins, and the remaining 9.22% (238) of drug targets were other organism proteins. In this study, the human drug targets were used to identify potential drug targets in *Plasmodium vivax*.

### 4.2. Heterogeneous Network Model for Human-Malaria Protein Association

A heterogeneous network usually consists of various networks and links between nodes on different networks [51]. In this work, the heterogeneous network was constructed based on the conjunction between a human protein interaction network and *Plasmodium vivax* malaria protein network by protein sequence similarities. Thus, the construction of the network is explained as follows.

Let $H=\{h_{1},\;h_{2},\;h_{3},\;\ldots ,\;h_{n}\}$ denote the n human protein nodes and $E_{hh}$ denote edges between human proteins in the human protein interaction network $G_{h}=(H,E_{hh},W_{hh})$, where $W_{hh}$ is its edge weights, which represent the protein interaction confidence scores from String database [31], Copenhagen, Denmark; Heidelberg, Germany; Lausanne, Switzerland. Similarly, the *Plasmodium vivax* malaria protein network is $G_{p}=(P,E_{pp},W_{pp})$, where $P=\{p_{1},\;p_{2},\;p_{3},\;\ldots ,\;p_{m}\}$ denotes the m *Plasmodium vivax* protein nodes, $E_{pp}$ denotes edges between malaria proteins, and $W_{pp}$ denotes its edge weights.

The connections between human proteins and malaria proteins are denoted as $E_{hp}$, which are edges of similar human-malaria protein pairs via BlastP search at NCBI, Bethesda, Maryland, United States. The weights on all these similar human-malaria protein pairs are initially assigned 1 and denoted by $W_{hp}$.

Finally, our heterogeneous network for our human-malaria protein association prioritization is represented as a graph:

$$G_{hp}=\left(G_{h}\cup G_{p},\;E_{hp},W_{hp}\right)$$

or

$$G_{hp}=\left(\left\{H,\;P\},\;E=\{E_{hh},E_{pp},E_{hp}\},\;W=\{W_{hh},W_{pp},W_{hp}\right\}\right)$$

This network model is well-known as a two-layer heterogeneous network model developed for drug target prediction [51]. Notice that this $G_{hp}$ has an incomplete graph with missing association edges between human and malaria proteins. To infer more association edges, the network propagation algorithm is then applied as explained in the next subsection.

### 4.3. Network Propagation Algorithm

As we have considered, this $G_{hp}$ is an incomplete graph with missing edges between human and malaria proteins. To find the hidden associations, we aimed to obtain new $E_{hp}$ and new $W_{hp}$ by employing the Heterogeneous Graph Based Inference (HGBI) algorithm [51], Manhattan, New York, United States. The HGBI is an iterative algorithm on a heterogeneous graph. Originally, the concept of this algorithm is based on the guilt-by-association principle [52,53] to infer new drug targets through existing relationships between similar drugs and similar targets. We employed this algorithm to find new potential associations between human and malaria proteins. The algorithm calculated updated weights by

(1) $$w(h_{i},p_{j})=\sum_{u\in H}\sum_{v\in P}w(h_{i},u)\cdot w(u,v)\cdot w(p_{j},v)$$

where w(a,b) is a weight between nodes a and b. This equation means that we can establish a new weight between a human-malaria protein pair by summarizing all paths over the three types of edges. This formulation is guaranteed to converge if the $W_{hh}$ and $W_{pp}$ are properly normalized [51] by

$$w(h_{i},h_{j})=\frac{w(h_{i},h_{j})}{\sqrt{\sum_{k=1}^{m}w(h_{i},h_{k})\sum_{k=1}^{m}w(h_{k},h_{j})}}$$

and

$$w(p_{i},p_{j})=\frac{w(p_{i},p_{j})}{\sqrt{\sum_{k=1}^{m}w(p_{i},p_{k})\sum_{k=1}^{m}w(p_{k},p_{j})}}$$

When the algorithm converges after some iterations, the information propagation is stabilized. Equation (1) can be formulated to a proper normalized matrix form as

(2) $$W_{hp}^{i+1}=\alpha (W_{hh}\times W_{hp}^{i}\times W_{pp})+(1-\alpha )W_{hp}^{0}$$

where α is a decay factor with its value between 0 and 1. In Equation (2), $W_{hp}^{0}$ is an initial weight matrix that we obtained from a sequence similarity search between human and malaria proteins.

### 4.4. Performance Measurement

To determine the performance of the algorithm, ten times ten-fold cross-validations were performed to evaluate the accuracy of the filtering by the heterogeneous network. The human proteins were randomly partitioned into ten parts. For each iteration, we hid weights of all human-malaria protein pairs in which their human proteins were included in the current part by setting the weights as zero, and the remaining pairs were used to recalculate the edge weights. When the iterative algorithm finished, the weights of the held-out human-malaria connections were collected. AUC or the area under the receiver operating characteristic (ROC) curve was used to measure the filtering performance. This scenario was performed ten times to obtain the average performance. In addition, the algorithm was performed with different parameter values α = 0.1, 0.2, 0.3, …, 0.9. We define the weight cutoff to determine whether a connection should be defined or not by maximizing the sensitivity and specificity. If a weight of a connection is greater than this cutoff value, the connection is established.

### 4.5. Association Module Clustering Algorithm

All obtained associations from the stabilized heterogeneous network integrated with known interactions from human protein interactions and *P. vivax* protein interactions were used to cluster proteins into groups via the ClusterONE algorithm [34], Egham Hill, Egham, United Kingdom. This algorithm is developed to introduce clustering methods based on overlapping neighborhood expansion for detecting potentially overlapping protein complexes from protein-protein interaction data. This algorithm consists of three steps. First, starting from a single seed vertex, vertices were added or removed to find groups by a greedy procedure to obtain high cohesiveness. The growth process is repeated from different seeds. Secondly, the extension of the overlapping between groups is quantified. The last step is to discard some complex candidates that contain less than three proteins or whose density is less than the threshold. We set the parameters for this algorithm with a minimal size of 3 and the node penalty of 2. The algorithm used single-pass merging method with a match coefficient similarity, and the overlap threshold was set as 0.8. Finally, we obtained 970 clusters, as shown in Supplementary Table S1.

### 4.6. Enrichment Test

Enrichment analysis was performed several times in this study. After the graph clustering, each of the human protein clusters were examined with the set of protein complexes and with the targets of the approved drugs. In addition, this was done to find pathways that our promising targets were enriched for. This enrichment test was performed using the one-sided Fisher’s exact test, and the obtained *p*-values were adjusted by Benjamini-Hochberg method for multiple-testing correction. The significance level cutoff of the adjusted *p*-values was set at 0.01. The clusters whose *p*-values were lower than the significance level were defined as enriched with either the protein complexes or the drug targets.

## 5. Conclusions

In conclusion, the use of a heterogeneous network provides us an opportunity to integrate various information into one graph and to discover the strength of relationships across different types of nodes, especially in our case between human host and malaria parasites. To quantify the direct interactions or associations between these two species is a complex and challenging task in wet-lab experiments. Computational identification of novel potential associations based on the heterogeneous network and the module clustering proposed in this study could serve as a challenging framework to prioritize and predict some human-malaria protein associations in advance. Thus, it helps us to better understand the complex interaction systems between host and parasite for prevention and treatment in the near future.

## Figures and Tables

**Table 1:** Network properties.

Properties	Human Network	Malaria Network	Initial State Heterogeneous Network	Stabilized Heterogeneous Network
Nodes	12,038	1787	13,825	13,825
Edges	313,359	11,477	334,361	6,422,644
Average degree	52.06	12.84	48.37	929.14
Transitivity	0.63	0.58	0.56	0.26
Eccentricity	9.45	9.83	9.56	4.72
Diameter	4.36	3.10	6.00	19.00

**Table 2:** The list of 7 clusters enriched in drug targets.

Cluster ID	The Number of Association Pairs	The Number of Known Drug Targets	The Number of Proteins Not Annotated as Drug Targets	The Ratio of Known Drug Targets	The Number of *P. vivax* Proteins
c1015 *	19	5	0	1.00	5
c1073 *	16	15	1	0.94	1
c1149	35,771	92	201	0.31	166
c1168	5373	56	50	0.53	66
c1185 *	88	13	5	0.72	7
c1199 *	256	15	10	0.60	11
c1208	26	6	7	0.46	2

* Clusters whose ratio of human proteins that were known drug targets to all human proteins in the cluster > 0.6.

**Table 3:** The list of pathways enriched with five promising human proteins in Cluster c1015.

Pathway ID	Pathway Name	*p*-Value (Benjamini–Hochberg Correction)
hsa00020	Citrate cycle (TCA cycle)	1.93 × 10^−10^
hsa01200	Carbon metabolism	1.13 × 10^−7^
hsa00190	Oxidative phosphorylation	4.12 × 10^−5^
hsa04932	Non-alcoholic fatty liver disease (NAFLD)	4.12 × 10^−5^
hsa05012	Parkinson disease	4.12 × 10^−5^
hsa05010	Alzheimer disease	5.84 × 10^−5^
hsa05016	Huntington disease	8.33 × 10^−5^
hsa04714	Thermogenesis	0.000147
hsa01100	Metabolic pathways	0.008858

**Table 4:** The list of drug names involved with the five promising human proteins in Cluster c1015.

Human Proteins	UniProt ID	UniProt Name	DrugBank ID	Drug Name	Associated Condition
*SDHA*	P31040	Succinate dehydrogenase [ubiquinone] flavoprotein subunit, mitochondrial	DB00139	Succinic acid	Dietary and Nutritional Therapies
*SDHA*	P31040	Succinate dehydrogenase [ubiquinone] flavoprotein subunit, mitochondrial	DB04657	Carboxin	Not Available
*SDHA*	P31040	Succinate dehydrogenase [ubiquinone] flavoprotein subunit, mitochondrial	DB09270	Ubidecarenone	Migraine
*SDHB*	P21912	Succinate dehydrogenase [ubiquinone] iron-sulfur subunit, mitochondrial	DB00139	Succinic acid	Dietary and Nutritional Therapies
*SDHC*	Q99643	Succinate dehydrogenase cytochrome b560 subunit, mitochondrial	DB00139	Succinic acid	Dietary and Nutritional Therapies
*SDHD*	O14521	Succinate dehydrogenase [ubiquinone] cytochrome b small subunit, mitochondrial	DB00139	Succinic acid	Dietary and Nutritional Therapies
*SDHD*	O14521	Succinate dehydrogenase [ubiquinone] cytochrome b small subunit, mitochondrial	DB00756	Hexachlorophene	Bacterial Infections
*SUCLG1*	P53597	Succinyl-CoA ligase [ADP/GDP-forming] subunit alpha, mitochondrial	DB00139	Succinic acid	Dietary and Nutritional Therapies

## Supplementary Materials

Supplementary materials can be found at https://www.mdpi.com/1422-0067/21/4/1310/s1.
