# Supplementary figures and images for: Heterogeneous Network Model to Identify Potential Associations Between Plasmodium vivax and Human Proteins
Source: Int J Mol Sci. 2020 Feb 15;21(4):1310. doi: 10.3390/ijms21041310 (PMC7072978; doi:10.3390/ijms21041310)

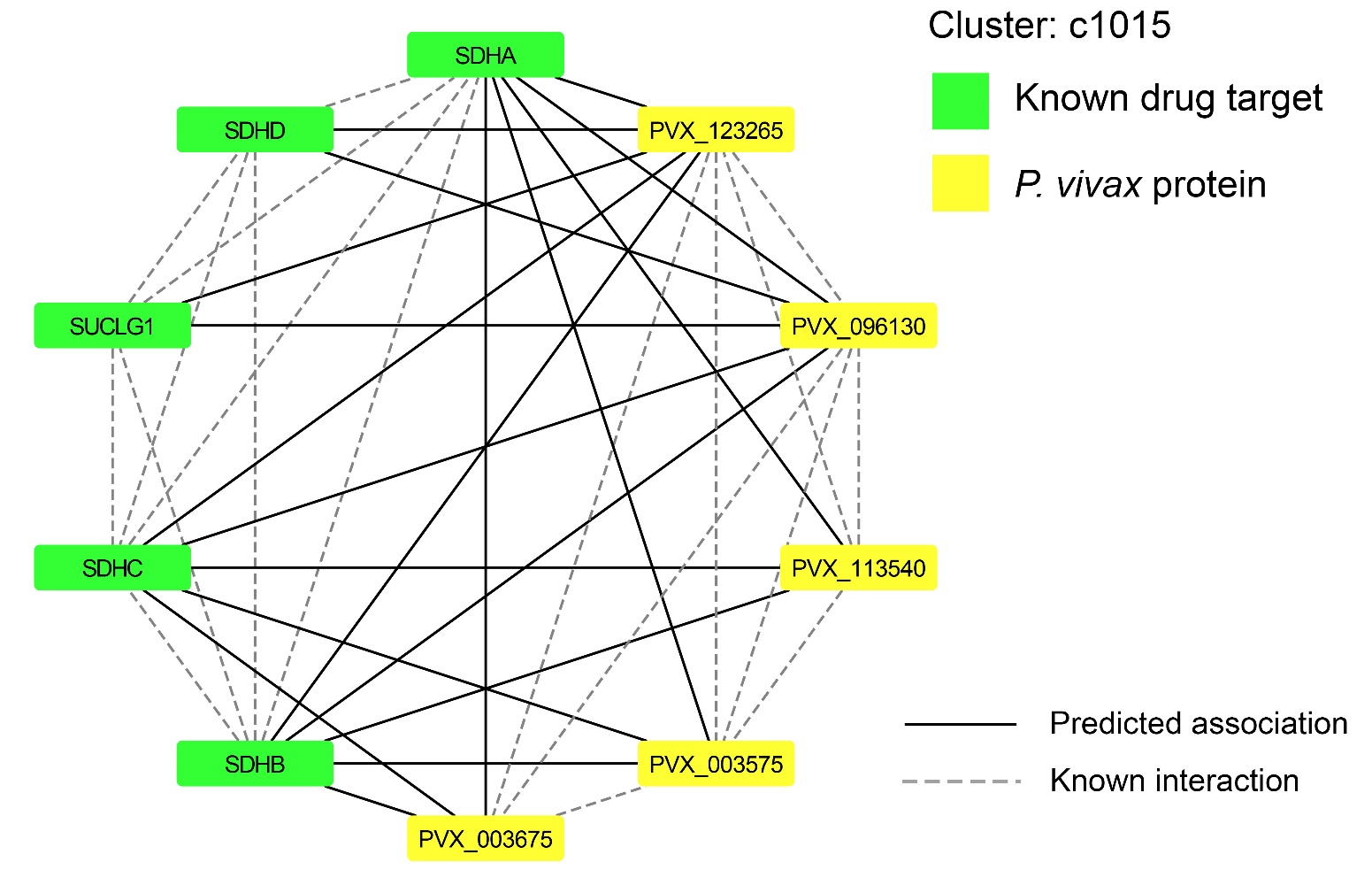


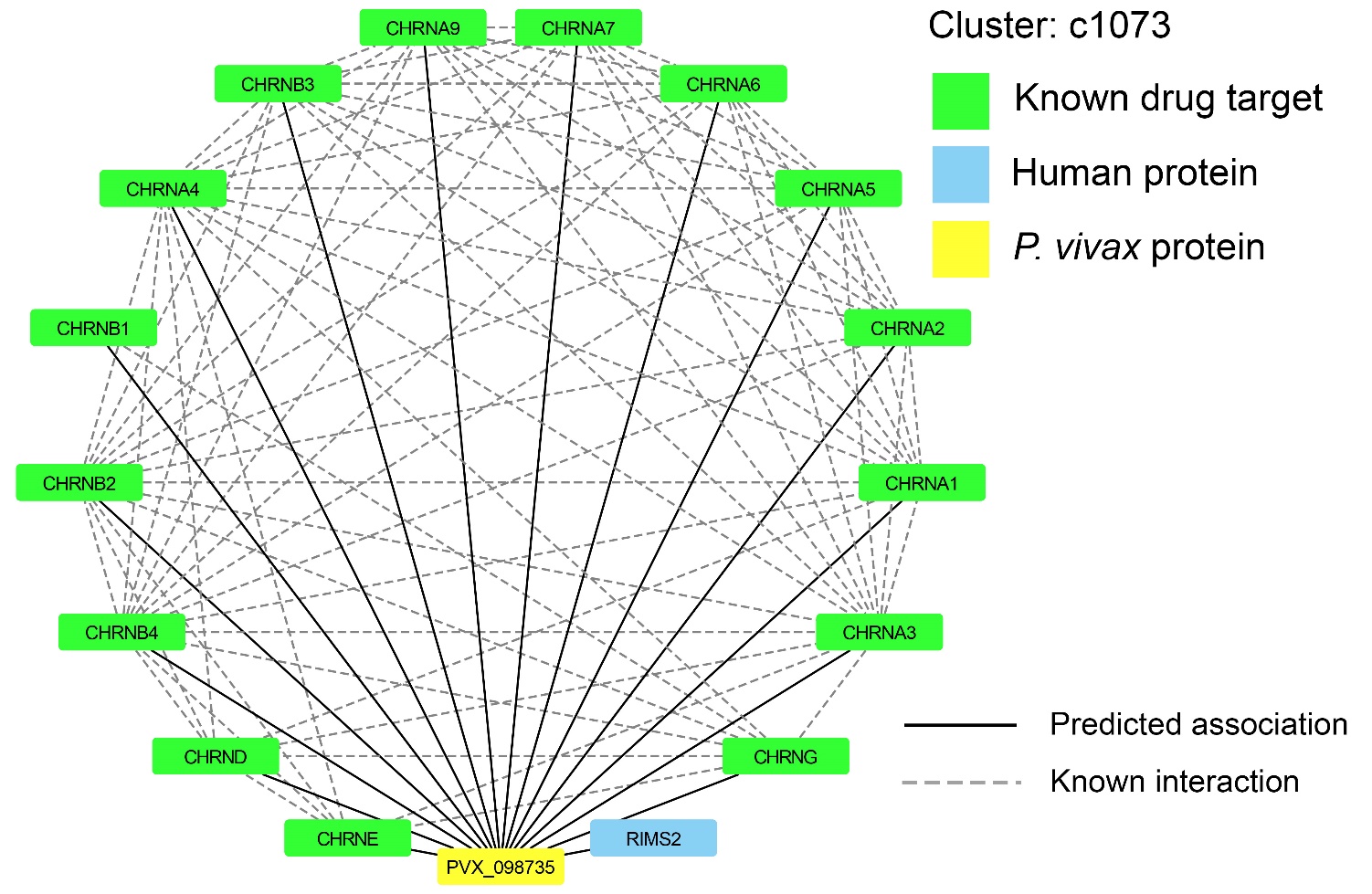


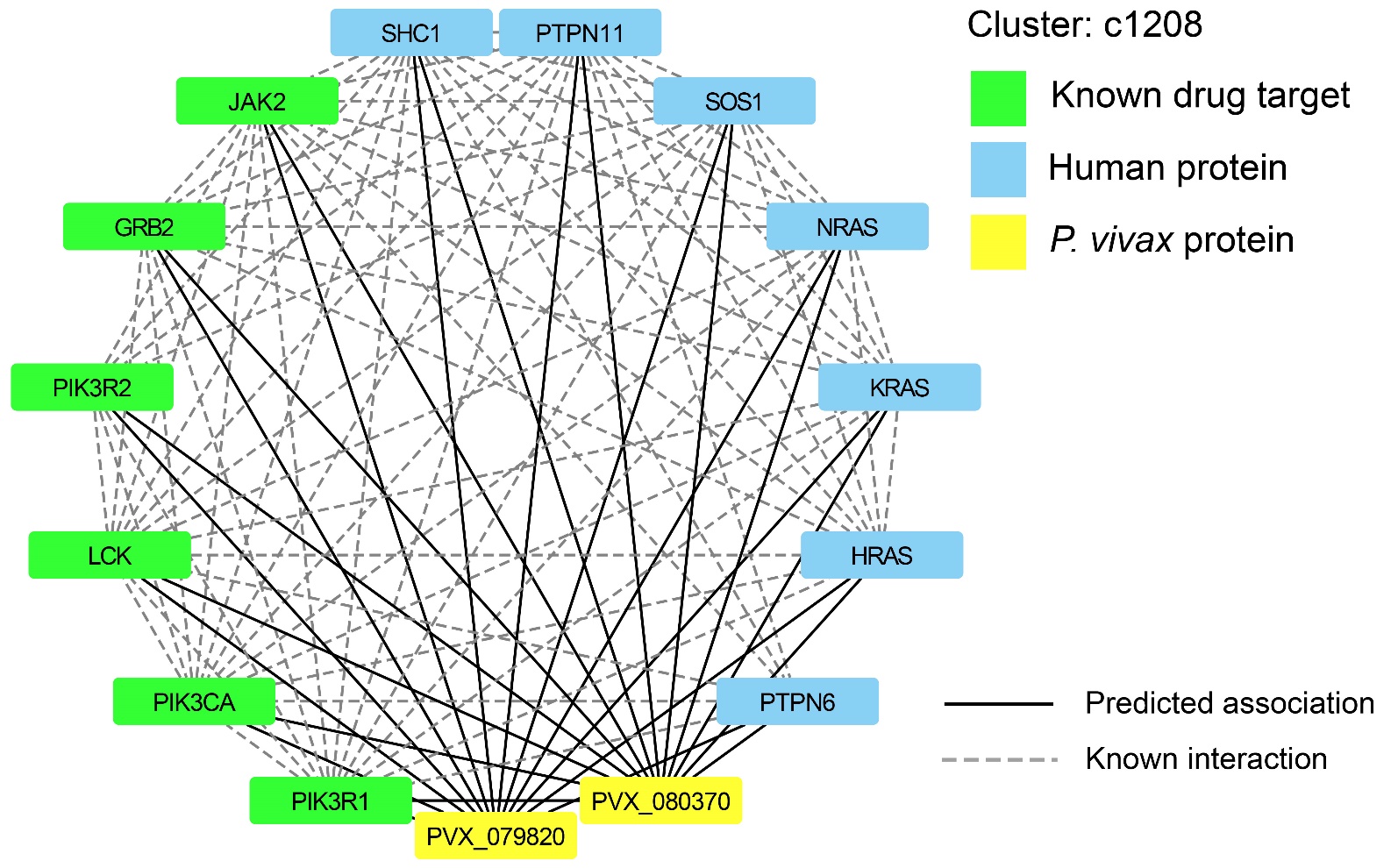


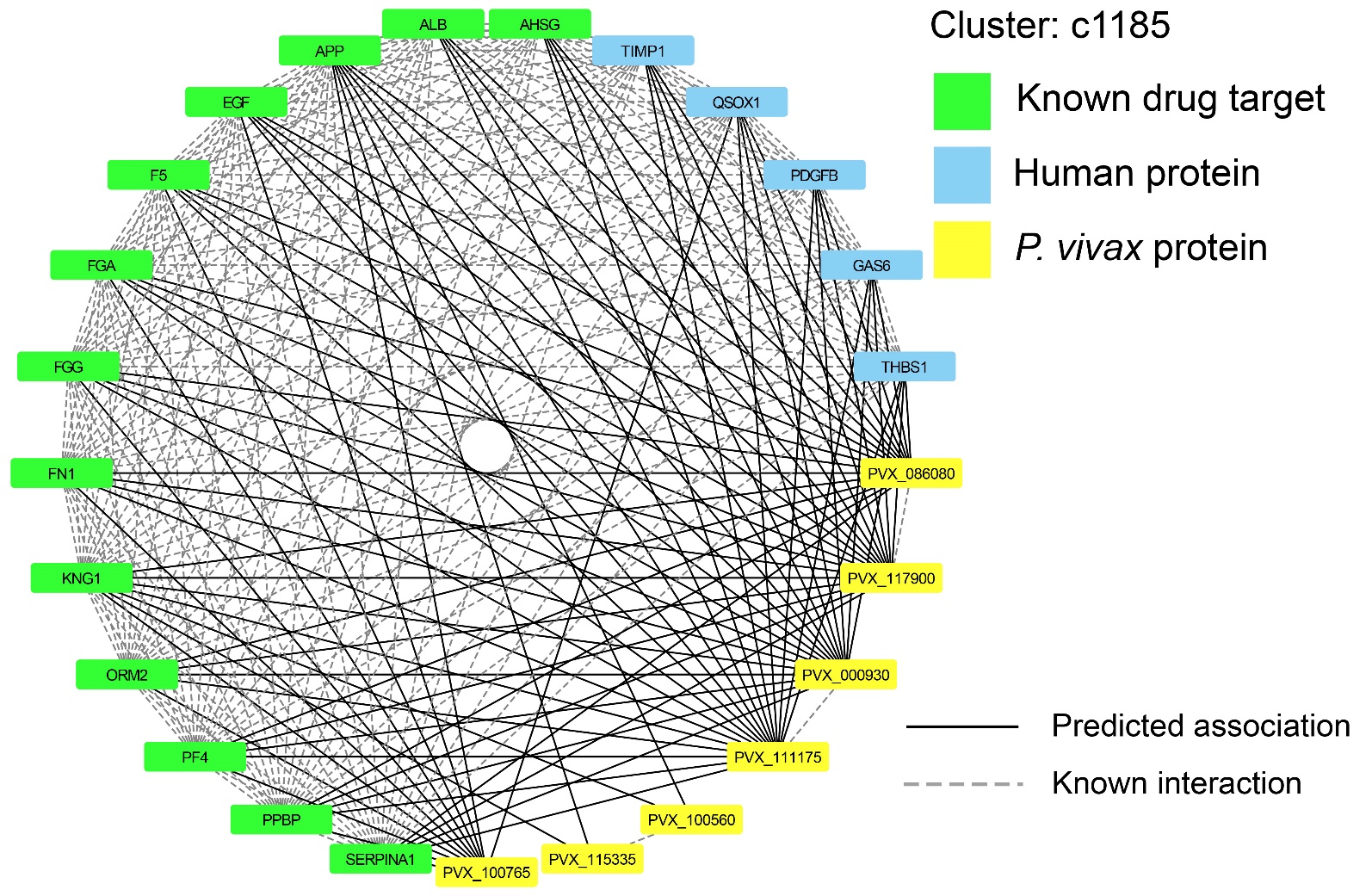


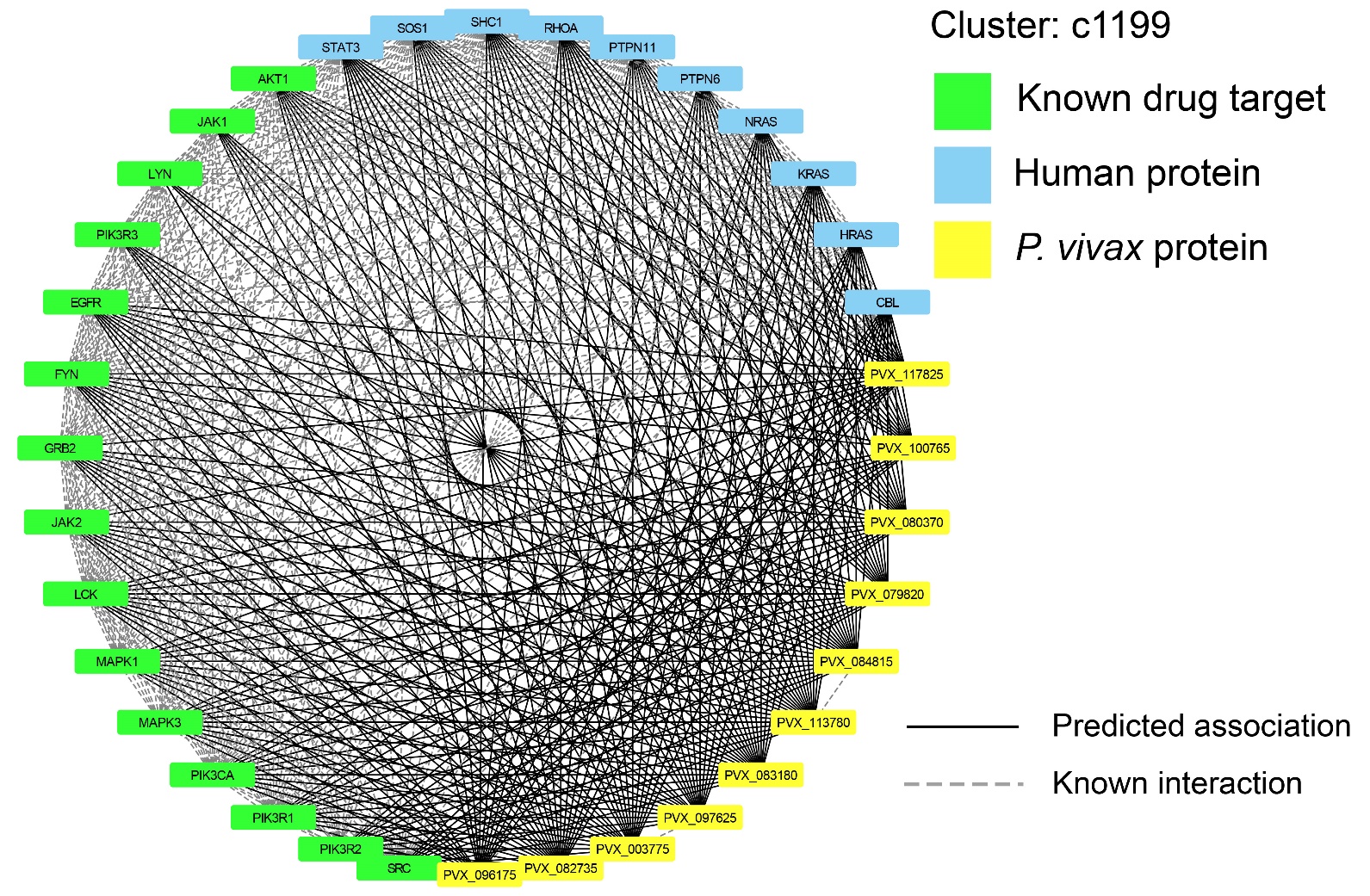


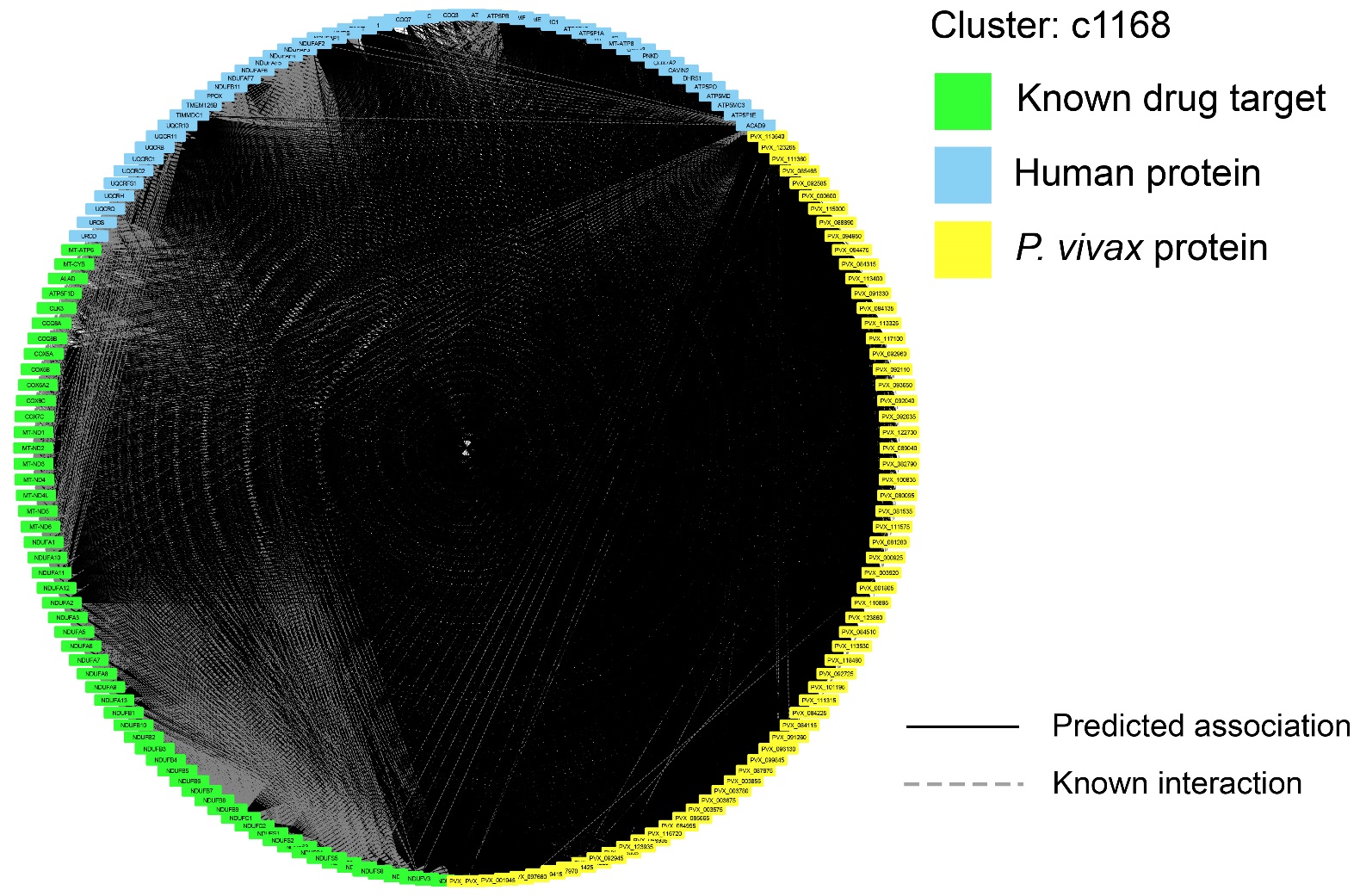


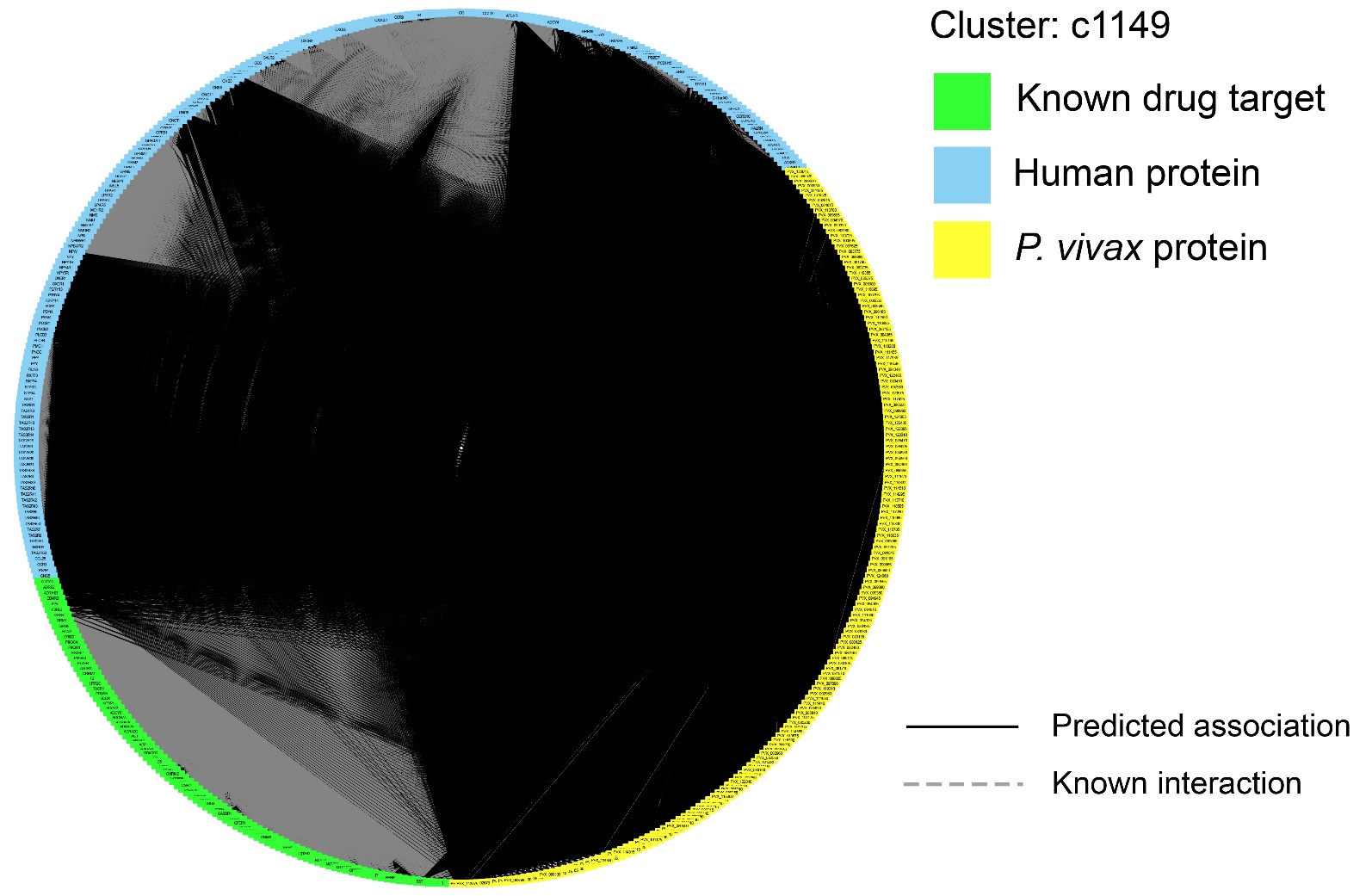

Supplement: Supplementary file 1 [file ijms-21-01310-s001.zip › NewSupplementary/SupplementaryFigureS7_Clusters.docx]
